# Associations between the Quality of the Residential Built Environment and Pregnancy Outcomes among Women in North Carolina

**DOI:** 10.1289/ehp.1103578

**Published:** 2011-12-02

**Authors:** Marie Lynn Miranda, Lynne C. Messer, Gretchen L. Kroeger

**Affiliations:** 1 Children’s Environmental Health Initiative, School of Natural Resources and Environment and Department of Pediatrics, University of Michigan, Ann Arbor, Michigan, USA; 2 Children’s Environmental Health Initiative, Nicholas School of the Environment, and; 3 Center for Health Policy and Inequalities Research, Duke Global Health Institute, Duke University, Durham, North Carolina, USA

**Keywords:** birth outcomes, built environment, health disparities

## Abstract

Background: The built environment, a key component of environmental health, may be an important contributor to health disparities, particularly for reproductive health outcomes.

Objective: In this study we investigated the relationship between seven indices of residential built environment quality and adverse reproductive outcomes for the City of Durham, North Carolina (USA).

Methods: We surveyed approximately 17,000 residential tax parcels in central Durham, assessing > 50 individual variables on each. These data, collected using direct observation, were combined with tax assessor, public safety, and U.S. Census data to construct seven indices representing important domains of the residential built environment: housing damage, property disorder, security measures, tenure (owner or renter occupied), vacancy, crime count, and nuisance count. Fixed-slope random-intercept multilevel models estimated the association between the residential built environment and five adverse birth outcomes. Models were adjusted for maternal characteristics and clustered at the primary adjacency community unit, defined as the index block, plus all adjacent blocks that share any portion of a line segment (block boundary) or vertex.

Results: Five built environment indices (housing damage, property disorder, tenure, vacancy, and nuisance count) were associated with each of the five outcomes in the unadjusted context: preterm birth, small for gestational age (SGA), low birth weight (LBW), continuous birth weight, and birth weight percentile for gestational age (BWPGA; sex-specific birth weight distributions for infants delivered at each gestational age using National Center for Health Statistics referent births for 2000–2004). However, some estimates were attenuated after adjustment. In models adjusted for individual-level covariates, housing damage remained statistically significantly associated with SGA, birth weight, and BWPGA.

Conclusion: This work suggests a real and meaningful relationship between the quality of the residential built environment and birth outcomes, which we argue are a good measure of general community health.

A relatively new area of attention in environmental public health is the impact of the built environment on health outcomes. In this article, we evaluated the quality of the built environment in the City of Durham, North Carolina (USA), and link neighborhood attributes to pregnancy outcomes in the same areas. We focus on pregnancy outcomes because poor birth outcomes are leading causes of neonatal mortality, as well as short- and long-term morbidity (Behrman and Butler 2007; Hack et al. 1995), and have been linked to adult disease end points, including diabetes, obesity, and cardiovascular disease (Barker et al. 1993; Osmond et al. 1993; Weiss et al. 2004).

Neighborhood sociodemographic conditions have long been associated with adverse reproductive outcomes. Modest, consistent associations have been noted for area-level deprivation and preterm birth (PTB) (Elo et al. 2009; Mason et al. 2009; O’Campo et al. 2008), low birth weight (LBW) (Nkansah-Amankra et al. 2010; Schempf et al. 2009), small for gestational age (SGA) (Elo et al. 2009; Farley et al. 2006), and neural tube defects (Grewal et al. 2009; Wasserman et al. 1998). Economic conditions, which are highly correlated with the quality of the built environment (Do et al. 2008), have been posited to influence PTB through differences in access to health care, the quality of available food, amount of green space, safe places for exercise, psychological stressors, and environmental pollutants (Diez-Roux 2003; Reagan and Salsberry 2005; Zeka et al. 2008).

Little prior work has explored built environment features and reproductive outcomes. Neighborhood characteristics may affect birth weight through effects on maternal behaviors, such as inappropriate weight gain (Laraia et al. 2007). Vinikoor et al. (2011) found that residence in census block groups characterized by high amounts of physical incivilities, which are physical markers of neighborhood degradation, was associated with adverse health behaviors and birth outcomes. Anxiety, depression, and general psychological distress have all been shown to increase with the number of housing problems (Evans et al. 2000; Wilkinson 1999). The built environment can also restrict residents’ physical activity (Saelens et al. 2003a, 2003b). Neighborhoods with low socioeconomic status suffer from such problems as dilapidated buildings and lack of exercise space and public services, which in turn negatively affect health (Acevedo-Garcia and Lochner 2003; Bullard 2000; Corburn 2004; Williams and Collins 2001). Features of the built environment may partially explain the long-observed associations between sociodemographic conditions and adverse health.

In this article, we describe the application of an innovative and user-friendly technology for documenting multiple features that shape the built environment. We surveyed approximately 17,000 residential tax parcels in central Durham on each of 50 individual variables during the summer of 2008. These data, collected from direct observation, were combined with tax assessor, public safety, and U.S. Census data to construct indices measuring different built environment domains. Potential associations between these indices and birth outcomes were assessed using multivariable regression analysis.

## Materials and Methods

**Figure 1:**
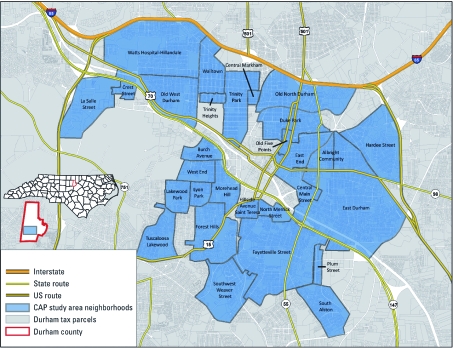
CAP study area, Durham, North Carolina.

*Study area.* The area covered by the Community Assessment Project (CAP) comprises 29 Durham neighborhoods (Figure 1). The neighborhoods included in the project area were selected to represent the socially and economically diverse urban core of Durham. The mean percentage of black non-Hispanic residents across Durham census tracts ranges from 4% to 98%, and the unemployment rate ranges from 1% to 45%.

*Data.* Durham County tax parcel data. We obtained tax parcel–level data for Durham County for the year 2008, which were assigned census location (blocks). Tenure (owner- or renter-occupied status, as defined by the U.S. Census Bureau 2010) was abstracted for parcels in the CAP study area (*n* = 17,239 parcels) by comparing the geographic address of the parcel to the owner’s address with an algorithm that allowed for minor errors in data entry and spelling. Addresses that matched were coded as owner-occupied residences; those that did not match were coded as renter occupied.

Built environment data. The built environment data were collected by trained field staff from the Children’s Environmental Health Initiative at Duke University (Durham, NC). Before data collection, a six-member field team attended sessions covering fundamental concepts of geographic information systems, use of the software package ArcGIS (version 9.3; ESRI, Redlands CA), and use of handheld Global Positioning System (GPS) units. During training, the team participated in trial assessments in predetermined areas to ensure variable coding, high interrater reliability, and GPS-unit competence.

Data collection occurred on foot, for approximately 10 weeks, between 0700 and 1330 hours, from late May to early August 2008. Individual tax parcels were assessed based on visibility from the public right-of-way.

To construct the data collection instrument, Children’s Environmental Health Initiative investigators identified observable variables that represented community concerns, based on direct conversations with community leaders, and supplemented the variable list with those represented in the literature (Caughy et al. 2001; Dustan et al. 2005; Raudenbush and Sampson 1999). Each parcel was assessed for 57 variables that categorized its land use; occupancy status; the physical condition of any buildings, yard, or property; the presence of nuisances; and security measures (as one marker of territoriality). The field team assessed public spaces for the presence of nuisances (e.g., broken glass, tree debris, litter) along roadways, curbs, sidewalks, and 2 feet into individual properties bordering the public space and recorded the presence and condition of sidewalks. Because certain nuisances were present both in public spaces and on properties, they were included in both variable lists, but public space and tax parcel nuisances were separate features. Residential, commercial, and other property types were similarly assessed. The resulting data collection instrument is available in the Supplemental Material (http://dx.doi.org/10.1289/ehp.1103578).

Crime data. We acquired crime data from the Crime Analysis Lab of the Durham Police Department. Data include the charge description and physical address of all reported crime incidents for 2006–2007. These data were geocoded and aggregated to the census block level. Each block was assigned a count for each crime type that occurred within that block: property, theft, vehicle, vice, violent, and total.

Vital records data. The North Carolina Detailed Birth Record databases for Durham County residents were obtained for the years 2004–2008. Maternal addresses were parcel geocoded to the 2008 Durham County Tax Assessor database (Durham, NC), with a geocoding match rate of 96%. Birth records were restricted to singleton, nonanomalous births occurring to women 15–44 years of age who self-identified as non-Hispanic white, non-Hispanic black, or Hispanic. Births were restricted to first through fourth births and to those occurring between 28 and 42 weeks’ gestation. Of the 16,783 live births that occurred to Durham County residents during this time period, 4,279 occurred to women who lived in the CAP study area and represent the analysis data set.

**Figure 2:**
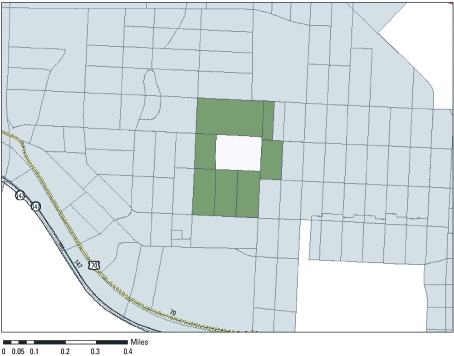
Illustration of PAC designation, City of Durham, North Carolina (USA).

*Community definition.* Because block-level data do not necessarily represent the most salient geography for effects on health outcomes, we constructed a new neighborhood unit, the primary adjacency community (PAC), to approximate each woman’s proximate community. A PAC includes the index block plus all adjacent blocks that share any portion of a line segment (block boundary) or vertex (Figure 2). We view the PAC as representing the immediate residential environment to which a woman is exposed while at home and during her trips near her residence.

The community data collection occurred independently of the assignment of outcomes to addresses. Teams rated each of the 17,239 residential parcels within 873 blocks. The number of blocks per PAC ranged from 2 to 20, with a mean ± SD of 7.2 ± 2.4 blocks.

*Built environment index development.* Although we recognize that the built environment includes the physical conditions of the home and other infrastructures that are created or modified by people (Srinivasan et al. 2003), this analysis is limited to residential properties.

To summarize the data in a meaningful way, we grouped the built environment variables into five domains: housing damage, property disorder, nuisances (in public spaces only), security measures, and vacancy. We examined the variables for shared features of the built environment, determined whether they contributed to the same latent construct, and grouped them into domains. Because this is the first tool to use such an exhaustive list of variables to characterize the built environment, little prior research aided in constructing the domains. However, we note that *a*) each domain is unique and does not contain variables that overlap with another domain, and *b*) the specificity of the domains will help disentangle features of the built environment most closely associated with health.

The CAP directly-observed parcel domains were housing damage (12 variables), property disorder (11 variables), security measures (6 variables), vacancy (3 variables), and nuisances (25 variables) (Table 1). Parcel-level data (the directly observed CAP data and the tenure data collected from the tax-parcel database) were summed at the block level to derive block-level counts of each variable for each domain. Block-level counts were divided by the total number of parcels in a given block to determine the block-level proportion of each variable for each domain. Crime data were obtained as counts of crime events per block.

**Table 1:** Variables for each community index for PAC units: CAP area, City of Durham, North Carolina (USA), 2008.

Characteristic	Mean ± SD (range)
Attribute	
Housing damage index (range, –0.48 to 3.42; α = 0.84)	
Boarded door	0.01 ± 0.02 (0–0.13)
Holes in walls	0.01 ± 0.02 (0–0.17)
Roof damage	0.02 ± 0.25 (0–0.26)
Chimney damage	0.001 ± 0.003 (0–0.04)
Foundation damage	0.002 ± 0.004 (0–0.03)
Entry damage	0.02 ± 0.02 (0–0.17)
Door damage	0.01 ± 0.02 (0–0.26)
Peeling paint	0.18 ± 0.12 (0–0.67)
>Fire damage	0.001 ± 0.01 (0–0.17)
Condemned	0.002 ± 0.01 (0–0.05)
Boarded windows	0.15 ± 0.26 (0–1.0)
Broken windows	0.06 ± 0.19 (0–1.0)
Property disorder index (range, –1.08 to 5.45; α = 0.83)	
Cars on lawn	0.04 ± 0.04 (0–0.22)
No grass	0.02 ± 0.02 (0–0.16)
Standing water	0.003 ± 0.01 (0–0.17)
Litter	0.23 ± 0.18 (0–1.0)
Garbage	0.07 ± 0.08 (0–1.0)
Broken glass	0.05 ± 0.09 (0–1.0)
Discarded furniture	0.03 ± 0.04 (0–0.33)
Discarded appliances	0.01 ± 0.01 (0–0.10)
Discarded tires	0.02 ± 0.03 (0–0.5)
Inoperable vehicle	0.02 ± 0.03 (0–0.16)
High weeds	0.11 ± 0.08 (0–1.0)
Security measures index (range, –1.71 to 5.72; α = 0.51)	
Security bars	0.04 ± 0.04 (0–0.38)
Barbed wire	0.02 ± 0.06 (0–1.0)
No trespassing sign	0.06 ± 0.07 (0–1.0)
Beware of dog sign	0.02 ± 0.02 (0–0.09)
Security sign	0.20 ± 0.12 (0–1.0)
Fencing	0.32 ± 0.15 (0–1.0)
Incidents per area	
Nuisance index (range, –1.05 to 6.52; α = 0.81)	
Shopping carts	0.03 ± 0.10 (0–1.0)
Total drug paraphernalia	0.01 ± 0.05 (0–0.38)
Inoperable car	0.04 ± 0.14 (0–1.5)
Food garbage	6.46 ± 5.64 (0–50.0)
Dog waste	0.16 ± 0.23 (0–2.0)
Tree debris	0.38 ± 0.40 (0–3.0)
Discarded furniture	0.14 ± 0.20 (0–2.0)
Discarded appliances	0.07 ± 0.12 (0–1.0)
Large trash	0.59 ± 0.58 (0–4.0)
Batteries	0.25 ± 0.33 (0–3.0)
Condoms	0.10 ± 0.19 (0–3.0)
Fallen wire	0.13 ± 0.19 (0–1.3)
Broken manhole cover	0.45 ± 0.48 (0–4.0)
Uncovered drain	0.04 ± 0.12 (0–2.0)
Cigarette butts	4.45 ± 3.50 (0–28.0)
Alcohol container	1.48 ± 3.50 (0–11.0)
Clothes	0.41 ± 0.53 (0–5.0)
Baby diapers	0.04 ± 0.10 (0–1.0)
Construction debris	0.09 ± 0.27 (0–4.0)
Deep holes	0.03 ± 0.10 (0–1.0)
Standing water	0.39 ± 0.69 (0–7.2)
Litter	14.11 ± 9.68 (0–112.0)
Broken glass	4.91 ± 4.15 (0–24.0)
High weeds	2.30 ± 1.76 (0–21.0)
Graffiti	0.003 ± 0.02 (0–0.25)
Crime count index (range, –0.66 to 11.36; α = 0.98)	
Violent crime	0.06 ± 0.78 (0.63–11.53)
Vice crime	0.06 ± 0.72 (0.67–10.24)
Vehicle crime	0.05 ± 0.68 (0.63–7.66)
Theft crime	0.07 ± 0.81 (0.63–12.28)
Property crime	0.07 ± 0.79 (0.63–9.92)
Area-level proportions	
Tenure (proportion renter)	Index mean ± SD = 0.00 ± 1.00 (range, –2.14 to 1.36)
Tenure (0 = owner; 1 = rented)	Variable mean ± SD = 0.55 ± 0.22
Vacancy (proportion vacant)	Index mean ± SD = 0.00 ± 1.00 (range, –0.82 to 3.96)
Vacancy (0 = occupied; 1 = not)	Variable mean ± SD = 0.16 ± 0.14
Cronbach’s α was used to assess the reliability of the standardized indices.

The block-level proportions were standardized (mean of 0, SD of 1) and summed at the PAC level to derive the PAC-level proportion of each variable. Proportions for each of the variables contained within a domain were added together and divided by the number of blocks contributing to that PAC to generate PAC-level domain-specific indices. For instance, the PAC-level security measures index = (standardized block-level proportion of security bars) + (standardized block-level proportion of barbed wire) + (standardized block-level no trespassing signs) + (standardized block-level beware of dog signs) + (standardized block-level security sign) + (standardized block-level fencing).

For each index, the more instances of an attribute, the higher its value (e.g., a PAC with a crime index of 5 has more crime than one with an index of 2). For tenure, higher index values represent more rental properties. Principal components analysis indicated that each variable weight was similar, so a simple summation of each variable produced comparable—and more easily interpretable—results.

*Outcome variable construction.* To be comparable with prior work on environmental contributors to birth outcomes (Miranda et al. 2009), five outcomes were constructed. Two continuous outcomes were considered: infant birth weight, reported in pounds and ounces and converted to grams, and infant birth weight percentile for a given week of gestational age (BWPGA). National Center for Health Statistics data on all U.S. live, singleton births for the years 2000–2004 (Centers for Disease Control and Prevention 2011) were used to construct sex-specific birth weight distributions for infants delivered at each gestational age. To calculate the distributions, we followed the methodology laid out by Alexander et al. (1996), except we used more recent and multiple years of data; because the multiple years of data effectively smoothed over outliers, their removal from the referent source was not necessary. Separately for male and female infants, we sorted the 19,261,916 births that occurred between 20 and 44 weeks’ within each week of gestation by birth weight in order to calculate birth weight percentiles. When multiple percentiles shared a single birth weight, the birth weight was assigned to the midpoint percentile. For each week of gestation at delivery, we used the reference distributions to determine the percentile for each reportable birth weight for both male and female infants. The full data set of births was linked by infant sex, gestational age, and birth weight to the list of percentiles for each reportable birth weight in order to construct the BWPGA outcome variable. This variable represents a continuous measure of fetal growth. We used the reference curves generated to define the 10th percentile cutoffs for SGA, as well as an innovative continuous measure of BWPGA. The BWPGA for each birth in our study population was calculated based on the appropriate sex-specific reference distribution for the corresponding gestational age. Three dichotomous outcomes were also constructed: PTB (< 37 weeks completed gestation), LBW (< 2,500 g birth weight), and SGA (< 10th percentile of birth weight for gestational age) (Alexander et al. 1996). For calculating PTB and SGA, the clinical estimate of gestational age was used.

Covariates. The adjusted models control for possible confounders to the built environment–birth outcome relationship that are reliably measured on the North Carolina birth record (Vinikoor et al. 2009): categorical maternal age (15–19, 20–24, 25–29, 30–34, 35–39, 40–44 years), categorical maternal education (< 12, 12, > 12 years), dichotomous birth order (first birth, > first birth), dichotomous marital status (married, not married), and infant sex (male, female). Categorical covariates were employed owing to some nonlinear relationships with the adverse birth outcomes (e.g., maternal age).

*Statistical analysis.* Counts (and percentages) of the categorical variables and means ± SDs of the continuous variables were calculated on the merged CAP–birth record data. Cronbach’s α was used to assess the reliability of the standardized indices. Both Pearson and Spearman correlation estimates were similar; therefore, we report the Pearson correlations among the indices. Crude and adjusted fixed-slope random-intercept multilevel models were estimated to predict the association between birth outcomes and PAC-level indices, adjusted for maternal covariates. Multilevel logistic regression models were used to estimate odds ratios (ORs) and 95% confidence intervals (CIs) for the three dichotomous outcomes (PTB, SGA, LBW), and β-coefficients (and 95% CIs) from linear regression models are reported for birth weight and BWPGA. The indices were entered singly (one index at a time) in their continuous form, and the statistical models were clustered at the block level; we used *p*-value < 0.05 for statistical significance. All analyses were conducted in Stata (version 11.0; StataCorp, College Station, TX).

## Results

*PAC descriptions.* The mean ± SD proportion of each variable comprising each index at both the block and PAC levels are listed in Table 1. The proportion of the CAP PACs with evidence of housing damage was small, but some heterogeneity (indicated by larger standard deviations) was noted. Similarly low proportions were noted for the variables representing property disorder (mean proportions ranged from 0.003 to 0.011). Among the variables that contributed to the security measures index, fencing and security signs were the most commonly observed by the raters (0.32 and 0.20, respectively), whereas barbed wire and beware of dog signs were rare. When nuisance items were counted, food garbage, cigarette butts, litter, broken glass, and high weeds were noted with frequency (ranging from 2.3 to 14.11 items per PAC). Theft and property crimes occurred most frequently (15.9 and 6.5 incidents per block, respectively), followed by violent crimes (3.6 crimes per block, on average). Approximately 55% of PAC residents were renters, and about 16% of PAC units were vacant. Each of the index α-values demonstrated considerable internal consistency, with α-values between 0.7 and 0.9 for all indices but security measures.

*Index correlations.* Correlations between the PAC-level indices were low (0.1–0.3) or moderate (0.4–0.6) (Table 2). The highest correlation occurred between property disorder and tenure (*r* = 0.6), and the lowest correlation was between security measures and nuisances (–0.002), suggesting that each of the indices are capturing distinct latent constructs.

**Table 2:** Index correlations at PAC level: CAP, City of Durham, North Carolina (USA), 2008.

Index	Housing	Property	Security	Tenure*^a^*	Vacancy	Crime	Nuisance*^b^*
Housing damage		1.00												
Property disorder		0.55		1.00										
Security measures		0.15		0.09		1.00								
Tenure*a*		0.43		0.61		–0.22		1.00						
Vacancy		0.50		0.55		–0.26		0.58		1.00				
Crime		0.11		0.30		–0.05		0.28		0.11		1.00		
Nuisance*b*		0.42		0.48		–0.002		0.34		0.27		0.52		1.00
**a**Renter- versus owner-occupied housing. **b**Potentially harmful or offensive detritus left after human activity in an area.														

*Associations between PAC indices and birth outcomes.* More than 40% of the women who resided in the CAP study area were black non-Hispanic, and nearly 40% were Hispanic. More than half the women were between 20 and 29 years of age at delivery. Of those who were ≥ 18 years of age, > 48% had less than a high school education. For most of the women, this was not their first pregnancy, and most were not married. Summary statistics on the women included in this analysis are presented in Table 3.

**Table 3:** Description of women residing in the CAP geographical area, Durham County, North Carolina (USA), 2004–2008.

Characteristic	Measure
Maternal race [*n* (%)]	
White non-Hispanic	739 (17.27)
Black non-Hispanic	1,850 (43.23)
Hispanic	1,690 (39.50)
Birth outcomes	
Dichotomous birth outcomes [*n* (%)]	
PTB	365 (8.53)
LBW	354 (8.27)
SGA	501 (11.71)
Continuous birth outcomes (mean ± SD)	
Birth weight (g)	3248.63 ± 581.95
BWPGA	43.08 ± 28.14
Maternal covariates	
Categorical maternal age, years [*n* (%)]	
15–19	705 (16.48)
20–24	1,388 (32.44)
25–29	1,046 (24.44)
30–34	730 (17.06)
35–39	335 (7.83)
40–44	75 (1.75)
Continuous maternal age, years (mean ± SD)	25.58 ± 6.07
Categorical maternal education, mothers ≥ 18 years of age [*n* (%)]	
> High school	1,141 (26.80)
High school	1,062 (25.95)
< High school	2,054 (48.25)
Dichotomous marital status [*n* (%)]	
Married	1,361 (31.81)
Not married	2,917 (68.19)
Parity [*n* (%)]	
First birth	1,681 (39.28)
≥ Second birth	2,598 (60.72)
Infant sex [*n* (%)]	
Female	2,106 (49.22)
Male	2,173 (50.78)
PAC mean index values*a* (mean ± SD)	
Housing damage	–0.06 ± 0.51
Property disorder	0.06 ± 0.63
Security measures	–0.07 ± 0.53
Tenure status	0.06 ± 0.70
Vacancy status	–0.07 ± 0.54
Total crime	1.31 ± 3.11
Total nuisances	0.42 ± 0.97
**a**Mean value for PAC indices in which women included in analysis resided.	

Residence in a PAC characterized by high amounts of housing damage and property disorder and more rental housing, vacant housing, and nuisances were associated with increased odds of PTB in unadjusted models (Table 4). After adjustment for probable confounders, associations with housing damage, property disorder, and vacancy status remained associated with elevated odds of PTB, but only tenure remained statistically significantly associated with increased odds of PTB among these women (OR = 1.23; 95% CI = 1.00, 1.52). Security measures and crime do not appear to be associated with PTB among this sample of women. Although total crime levels are presented here, no associations between birth outcomes and crime of any type were observed (data not shown).

**Table 4:** Multilevel logistic regression results of continuous PAC-level indices: unadjusted and adjusted models representing the effect of one-unit change in continuous built environment exposures on dichotomous birth outcomes [OR (95% CI)].

Dichotomous outcomes										
PTB			SGA			LBW		
Index	Unadjusted*^a^*	Adjusted*^b^*	Unadjusted*^a^*	Adjusted*^b^*	Unadjusted*^a^*	Adjusted*^b^*
Housing damage		1.36 (1.10, 1.67)		1.21 (0.98, 1.49)		1.49 (1.26, 1.75)		1.27 (1.08, 1.51)		1.33 (1.09, 1.62)		1.07 (0.88, 1.31)
Property disorder		1.49 (1.22, 1.82)		1.20 (0.97, 1.50)		1.49 (1.27, 1.75)		1.13 (0.95, 1.35)		1.55 (1.28, 1.87)		1.12 (0.93, 1.35)
Security measure		0.93 (0.72, 1.20)		0.91 (0.71, 1.15)		1.05 (0.86, 1.29)		0.99 (0.81, 1.20)		1.09 (0.86, 1.37)		1.01 (0.83, 1.24)
Tenure status		1.39 (1.14, 1.69)		1.23 (1.00, 1.52)		1.20 (1.03, 1.40)		0.97 (0.83, 1.15)		1.35 (1.11, 1.63)		1.08 (0.91, 1.29)
Vacancy status		1.40 (1.12, 1.75)		1.20 (0.96, 1.50)		1.49 (1.23, 1.80)		1.19 (0.99, 1.44)		1.36 (1.09, 1.68)		1.05 (0.86, 1.28)
Total crime		1.02 (0.94, 1.10)		1.00 (0.94, 1.07)		1.02 (0.97, 1.07)		1.00 (0.96, 1.05)		1.01 (0.94, 1.07)		0.99 (0.95, 1.04)
Nuisance count		1.23 (1.06, 1.42)		1.10 (0.95, 1.27)		1.18 (1.02, 1.34)		1.02 (0.91, 1.15)		1.20 (1.04, 1.39)		1.01 (0.90, 1.14)
**a**Unadjusted models. **b**Models adjusted for categorical maternal race (non-Hispanic white, non-Hispanic black, Hispanic), age (15–19, 20–24, 25–29, 30–34, 35–39, 40–44 years), education (< 12, 12, > 12 years), dichotomous birth order (first birth, > first birth), and marital status (married, not married) and for infant sex (male, female).												

Similar to the results for PTB, residence in a PAC with higher index values of housing damage, property disorder, tenure status, vacancy status, and nuisances was associated with increased odds of SGA in the unadjusted models (Table 4). After adjustment for confounders, most of these estimates could no longer be distinguished from null findings, except for housing damage, which remained statistically significant (OR = 1.27; 95% CI: 1.08, 1.51). No association between SGA and security measures or crime was observed in these models.

For the LBW outcome, residence in a PAC with higher index values of housing damage, property disorder, tenure status, vacancy status, and nuisances was associated with increased odds of LBW, but these estimates were close to the null in the adjusted models (Table 4). No association with LBW was observed for security measures or crime in unadjusted or adjusted models.

Women who live in PACs with more housing damage delivered infants of lower average birth weights and BWPGA, in both unadjusted and adjusted models (Table 5). Birth weights and BWPGA were also lower for women residing in PACs with more property disorder, rentals (tenure status), vacancies, and nuisances, but these associations were attenuated after adjustment for covariates. For instance, after adjustment, a 1-unit increase in housing damage was associated with a 50-g decrement in birth weight (95% CI: –89.21, 11.34) and a 2.2 percentile decrement in BWPGA (95% CI: –3.96, –0.42). There appeared to be no relationship between security measures or crime and either outcome.

**Table 5:** Multilevel linear regression results of continuous PAC-level indices; unadjusted and adjusted models representing the effect of one-unit change in continuous built environment exposures on continuous birth outcomes [β (95% (CI)].

Continuous outcomes						
Birth weight			BWPGA		
Index	Unadjusted*a*	Adjusted*b*	Unadjusted*a*	Adjusted*b*
Housing damage		–119.14 (–162.35, –75.93)		–50.27 (–89.21, –11.34)		–5.52 (–7.50, –3.55)		–2.19 (–3.96, –0.42)
Property disorder		–130.13 (–167.26, –93.00)		–26.77 (–62.28, 9.73)		–5.65 (–7.35, –3.96)		–0.72 (–2.37, 0.92)
Security measure		–0.34 (–51.48, 50.80)		6.16 (–35.01, 47.34)		–0.31 (–2.59, 1.97)		0.07 (–1.72, 1.86)
Tenure status		–104.20 (–138.43, –69.96)		–25.71 (–58.57, 7.15)		–4.13 (–5.69, –2.56)		–0.10 (–1.55, 1.35)
Vacancy status		–119.24 (–161.82, –76.67)		–34.86 (–73.50, 3.78)		–5.48 (–7.41, –3.55)		–1.44 (–3.18, 0.29)
Total crime		–15.92 (–34.44, 2.60)		3.03 (–10.06, 16.12)		–0.64 (–1.42, 0.13)		0.08 (–0.39, 0.56)
Nuisance count		–73.44 (–102.54, –44.34)		–7.03 (–33.49, 19.42)		–2.98 (–4.29, –1.66)		0.11 (–1.05, 1.26)
**a**Unadjusted models. **b**Models adjusted for categorical maternal race (non-Hispanic white, non-Hispanic black, Hispanic), age (15–19, 20–24, 25–29, 30–34, 35–39, 40–44 years), education (< 12, 12, > 12 years), dichotomous birth order (first birth, > first birth), and marital status (married, not married) and for infant sex (male, female).								

## Discussion

Increasing attention has been paid to the potential role of the built environment in shaping health outcomes and determining health disparities. We found that five of the built environment indices (housing damage, property disorder, tenure status, vacancy status, and nuisances) were associated with each of our five outcomes (PTB, SGA, LBW, continuous birth weight, and BWPGA) before adjustment, but some of the estimates were attenuated after adjustment for maternal characteristics. After adjustment, housing damage remained statistically significantly associated with SGA, birth weight, and BWPGA. Thus, in this exploratory work, we found specific, measurable features of the built environment to be consistently associated with increased risk of adverse birth outcomes in unadjusted models and housing damage to be associated with SGA, birth weight, and BWPGA after confounder adjustment.

The built environment is multidimensional, which makes it difficult to characterize any given area as having a “good” or “bad” built environment. In this article, we describe a geographically based approach to efficiently inventory the built environment at a highly resolved geographic scale, as well as methods for combining built environment data into seven different domains. These domains represent latent community health status and are conceptually and empirically distinct from each other. Housing damage and property disorder both capture the physical state of a property but focus on separate features. Security measures also describes a parcel but describe measures taken by residents or land owners to delineate property boundaries and protect ownership. Tenure and vacancy describe who, if anyone, is occupying a parcel, and crime captures the quantity and type of crime occurring within a resident’s community. Nuisances capture the detritus that results from human activity and reflects both treatment and monitoring of the local environment.

Despite limited research assessing the association between the built environment and health outcomes, our findings are consistent with prior work. Both Vinikoor et al. (2011) and Laraia et al. (2007) found that territoriality, assessed using similar—but not identical—security indicators, was not associated with maternal health behaviors or outcomes, so finding no effect of our security measures index was not surprising. Finding no effect for PAC crime was somewhat unexpected, given that prior research in an adjacent county (Wake) found that crime was associated with increased odds of PTB (Messer et al. 2006). Given limited prior research, literature assessing possible mechanisms from the built environment to health outcomes is scarce. Built environment features may not represent actual exposures per se but rather capture some underlying latent conditions to which women are exposed in their residential environment conferring increased odds of adverse birth outcomes. It may also be that externally observed housing damage reflects damage/adverse exposures inside the home as well. These and other mechanisms will need to be explored in this rapidly expanding line of research.

Although novel, this work is not without limitations. Birth records have been criticized for data quality limitations, but we limited our analyses to data elements with demonstrated reliability (Vinikoor et al. 2009). Additionally, we have no information on how long women resided in our constructed PACs before delivering. However, research on pregnant women in Texas revealed that although 32% of mothers changed residence between conception and birth, very few (4%) actually moved to a new U.S. census block group (Nuckols et al. 2004). In addition, Canfield et al. (2006) determined the misclassification associated with residential mobility to be nondifferential, which biases the findings toward the null value and affects cases and noncases similarly. Further, there is a slight temporal mismatch between exposure collection and the birth outcomes, but we assessed temporal trends in both birth outcomes and change in neighborhood characteristics, and they do not appear to account for the results we report here. We also chose to omit maternal smoking as a model covariate because research suggests that, among nonsmokers, residing in less habitable neighborhood was associated with smoking uptake (Burgess 2006; Frohlich et al. 2002), and neighborhood norms may contribute to smoking uptake (Ahern et al. 2009); therefore, smoking may lie on the causal path between neighborhoods and birth outcomes, and to include it in models would likely adjust away part of the effect we are trying to observe.

One important strength of this work is our unique, thorough, and easily replicable data collection methodology. By collecting these directly-observed data using a geospatial approach, we were able to audit a large number of tax parcels in a relatively short amount of time and with modest resources. Further, we collected data on geographically contiguous communities, not just a sample of streets, resulting in complete exposure ascertainment. We extend prior built environment work by breaking physical incivilities into its constituent parts (housing damage, property disorder, and nuisances) and adding additional indices for consideration. Further, our exposure assessment was independent of the outcome.

Despite its limitations, this work suggests a robust relationship between the quality of the built environment and birth outcomes, which we argue is a good measure of general community health. Expanding the scope of environmental health research beyond traditional measures of pollutant exposure to include the built environment is critical to more fully understanding environmental etiologies of human disease.

## Supplemental Material

(193 KB) PDF
