# Supplementary material for: Associations between the Quality of the Residential Built Environment and Pregnancy Outcomes among Women in North Carolina
Source: Environ Health Perspect. 2011 Dec 2;120(3):471–7. doi: 10.1289/ehp.1103578 (PMC3295337; doi:10.1289/ehp.1103578)
Supplement: (193 KB) PDF [file ehp.1103578.s001.pdf]

Supplemental Material

Associations Between the Quality of the Residential Built Environment and Pregnancy Outcomes Among Women in North Carolina

Marie Lynn Miranda, Lynne C. Messer, and Gretchen L. Kroeger

Table of Contents

Table 1. Community Assessment Project Tool: Variable List

Parcel-level Variables

Field	Values
Property Type	
	Residential
	Commercial
	Community
	Government
	Faith
	Empty Lot
	Residential-Commercial
	Other Mix Type
	Parking Lot
Property Status	
	Occupied
	Unoccupied
	Demolished
Residential Type	
	Single Family
	Apartment Complex, 3-6 units
	Apartment Complex, 6 or more units
	Duplex
	Care Facility
	Senior Housing
	Multi-Address Home
	Mobile Home
	Other Mix Type
Other Residential Type Description	
Community Type	
	Alcohol and Drug Treatment Center
	Cemetery
	Community Recreation/Cultural Center
	Hospital
	Military Service Club
	Park or Playground
	Public Health Care/Clinic
	School K-12
	School, University
	Other
Other Community Type Description	
Empty Lot Type	
	Residential
	Commercial

Field	Values
Commercial Type	
	Business Office
	Child Care Center
	Medical Office
	Dental Office
	Industrial Building
	Restaurant, Family
	Restaurant, Drive-up
	Hair Salon/Barber Shop
	Auto Repair Shop
	Bar/Club
	Liquor Store
	Adult Merchandise
	Grocery Store
	Gas/Convenience
	Pawn
	Gun Shop
	Private Gym
	Law Office
	Bail Bond
	Utility/Gas/Electric Plant
	Day Laborer Office
	Check-Cashing/Money-Lending
	Tienda
	Retail
	Other
	Vacant
Other Commercial Type Description	
Faith Type	
	Church
	Mosque
	Synagogue
	Temple
	Other
Other Faith Type Description	
Residential-Commercial Type	
	Day Care
	Hair Salon/Barber Shop
	Other Residential-Commercial Type
Other Residential-Commercial Type	
Other Mix Type Description	

Parcel-level Variables, Continued

Field	Values	
Parking Lot Type		
	Residential	
	Commercial	
	Community	
	Government	
	Faith	
	Empty Lot	
	Residential-Commercial	
	Other Mixed Type	
Broken Windows		
	0	*No*
	1-10	*Yes*
Boarded Windows		
	0	*No*
	1-10	*Yes*
Boarded Door		
	0	*No*
	1	*Yes*
Holes in Walls		
	0	*No*
	1	*Yes*
Roof Damage		
	0	*No*
	1	*Yes*
Residential Chimney Damage		
	0	*No*
	1	*Yes*
Foundation Damage		
	0	*No*
	1	*Yes*
Residential Front Entry Type		
	Stoop	
	Porch	
Residential Front Entry Damage		
	0	*No*
	1	*Yes*
Door Damage		
	0	*No*
	1	*Yes*
Peeling Paint		
	0	*No*
	1	*Yes*

Field	Values	
Peeling Paint Area		
	Windows	
	Siding	
	Both	
Fire Damage		
	0	*No*
	1	*Yes*
Other Condition		
Condemned		
	0	*No*
	1	*Yes*
Eviction Notice		
	0	*No*
	1	*Yes*
Padlocked		
	0	*No*
	1	*Yes*
Residential Driveway		
	0	*No*
	1	*Yes*
Residential Cars on Lawn		
	0	*No*
	1	*Yes*
Residential Garden		
	0	*No*
	1	*Yes*
Residential Greenery		
	0	*No*
	1	*Yes*
Residential No Grass		
	0	*No*
	1	*Yes*
Standing Water		
	0	*No*
	1	*Yes*
Litter		
	0	*No*
	1	*Yes*
Garbage		
	0	No
	1	Yes

Field	Values	
Broken Glass		
	0	*No*
	1	*Yes*
Discarded Furniture		
	0	*No*
	1	*Yes*
Discarded Appliances		
	0	*No*
	1	*Yes*
Discarded Tires		
	0	*No*
	1	*Yes*
Inoperable Vehicle		
	0	*No*
	1	*Yes*
High Weeds/Grass		
	0	*No*
	1	*Yes*
Graffiti		
	0	*No*
	1	*Yes*
Other Nuisance		
Fencing		
	0	*No*
	1	*Yes*
Fence Damage		
	0	*No*
	1	*Yes*
Fence Material		
	Wood	
	Chain-link	
	Wire	
	Vinyl	
	Brick	
	Iron	
	Combination	
	Other	
Fence Area		
	Entire	
	Backyard	
	Front yard	
	Other	

Parcel-level Variables, Continued

Field	Values	
Security Bars		
	0	No
	1	Yes
Barbed Wire		
	0	No
	1	Yes
No Trespassing Sign		
	0	No
	1	Yes
For Sale Sign		
	0	No
	1	Yes
For Rent Sign		
	0	No
	1	Yes
Residential Other Sign		
Dog		
	0	No
	1	Yes
Window AC Unit		
	0	No
	1	Yes
Home Repair		
	0	No
	1	Yes
New Home Construction		
	0	No
	1	Yes
Comments		
Rater		

Nuisances

Field	Values	
Graffiti		
	0	No
	1	Yes
Broken Glass		
	0	No
	1	Yes
Shopping Cart		
	0	No
	1	Yes
Litter		
	0	No
	1	Yes
Drug Paraphernalia		
	0	No
	1	Yes
Inoperable Vehicle		
	0	No
	1	Yes
High Weeds/Grass		
	0	No
	1	Yes
Dog Waste		
	0	No
	1	Yes
Tree Debris		
	0	No
	1	Yes
Standing Water		
	0	No
	1	Yes

Nuisances, Continued

Field	Values	
Discarded Furniture		
	0	No
	1	Yes
Discarded Appliances		
	0	No
	1	Yes
Discarded Tires		
	0	No
	1	Yes
Large Trash		
	0	No
	1	Yes
Batteries		
	0	No
	1	Yes
Condoms		
	0	No
	1	Yes
Fallen Wires		
	0	No
	1	Yes
Broken Meter Cover		
	0	No
	1	Yes
Uncovered Storm Drain		
	0	No
	1	Yes
Cigarette Boxes or Butts		
	0	No
	1	Yes

Nuisances, Continued

Field	Values	
Alcohol Container		
	0	No
	1	Yes
Food Garbage		
	0	No
	1	Yes
Clothes or Shoes		
	0	No
	1	Yes
Baby Diapers		
	0	No
	1	Yes
Construction Debris		
	0	No
	1	Yes
Deep Hole		
	0	No
	1	Yes
Other		
Comments		
Rater		

Supplemental Material, Table 1
